# Total hip arthroplasty in Italy: an observational, population-based study on surgical volume growth from 2001 to 2023 and forecasts until 2050 with six different statistical models

**DOI:** 10.1186/s10195-025-00893-4

**Published:** 2025-12-04

**Authors:** Enrico Ciminello, Adriano Cuccu, Emilio Romanini, Michele Venosa, Gianpiero Cazzato, Gabriele Tucci, Filippo Boniforti, Luca Carpanese, Tiziana Falcone, Stefania Ceccarelli, Paola Ciccarelli, Marina Torre

**Affiliations:** 1 Italian Implantable Prostheses Registry (RIPI), Italian National Institute of Health, Viale Regina Elena 299, 00161 Rome, Italy; 2 Department of Statistical Sciences, “La Sapienza” University of Rome, Piazzale Aldo Moro 5, 00185 Rome, Italy; 3 RomaPro, Polo Sanitario San Feliciano, Via Mattia Battistini 44, 00167 Rome, Italy; 4 Italian Working Group On Evidence-Based Orthopaedics, GLOBE, Via Nicola Martelli 3, 00197 Rome, Italy; 5 Ospedale Dei Castelli, Ariccia, 00040 Rome, Italy; 6 Fondazione Istituto G. Giglio, 90015 Cefalù, Italy

**Keywords:** Hip replacement, Arthroplasty, Forecasting, Projections, Trends, Epidemiology

## Abstract

**Background:**

The number of total hip arthroplasty (THA) procedures has been steadily increasing worldwide, driven by aging population, improvements in surgical techniques and implant design. This study aimed to analyze the temporal trends of elective THA in Italy since 2001–2023 and forecast THA volumes up to 2050 to provide insights for healthcare planning.

**Materials and methods:**

International Classification of Diseases, 9th Revision, Clinical Modification (ICD9-CM) coding system was used to extract records of interest (elective THA) from the Italian National Hospital Discharge Record database. Six statistical models were applied to forecast future THA volumes: logistic regression; Poisson regression; logarithmic regression; inverse/power regression; Poisson log-normal regression; and hierarchical Poisson regression with temporal effects (HPTE). Model performances were assessed by using error metrics and internal validation on the basis of a rolling-origin approach. An out-of-sample validation was conducted to ensure a robust assessment of forecasting reliability. THA volume forecasts were provided with 95% prediction intervals.

**Results:**

A total of 1,318,400 records for primary elective THAs performed in Italy since 2001–2023 were analyzed. The number of THAs increased by approximately 80%, rising from 68.270 in 2001 to 122.777 in 2023. Among the tested models, HPTE generally showed the best fitting and forecasting performances. By using the HPTE model, the forecasts showed an increase in THA volumes up to a maximum rate ratio (RR) of 1.3 (PI_95%_: 1.1–1.4) in terms of RR in 2036, then decreasing to a RR equal to 1.2 (PI_95%_: 1.1–1.4) by 2050 with respect to 2019.

**Conclusions:**

Our findings forecast a steady increase between 10% and 40% in THA, driven by demographic and epidemiological trends. These projections are essential for anticipating future surgical demand and guiding healthcare system planning. Without adequate investment and strategic planning, rising volumes may strain service capacity and sustainability.

*Level of evidence*: population based study, level 1 evidence.

**Supplementary Information:**

The online version contains supplementary material available at 10.1186/s10195-025-00893-4.

## Introduction

Hip osteoarthritis has become increasingly prevalent over the past decades, primarily driven by population aging and rising obesity rates [1–4]. Advances in surgical techniques and implant design have improved long-term patient outcomes, allowing total hip arthroplasty (THA) to be offered to younger and more active individuals affected by hip osteoarthritis [5, 6]. Owing to its success in restoring mobility and quality of life, THA has been described as ‘the operation of the century’ [5]. As a result, the annual number of hip replacements has steadily increased in developed countries and is expected to continue rising, placing a growing economic and organizational burden on healthcare systems [7–11].

Accurately forecasting the future demand for hip arthroplasty is crucial to support strategic planning and resource allocation, particularly in Italy, which has the highest proportion of elderly citizens (aged > 65) in Europe (24,3% in 2024) [12, 13]. At the time being, a published paper explored total knee arthroplasty trend in Italy and provided a projection of surgery volumes until 2050 [14], but an analysis targeting hip arthroplasty was not yet carried out.

The aim of this study is to analyze the epidemiology of elective hip arthroplasty in Italy from 2001 to 2023 and forecast surgery volumes up to 2050 using a range of statistical models.

## Material and methods

This observational, population-based study was carried out according to the Strengthening the Reporting of Observational Studies in Epidemiology (STROBE) guidelines provided by the Enhancing the Quality and Transparency of Health Research (EQUATOR) Network [15]. Moreover, the study passed all checks of the REporting of studies Conducted using Observational Routinely-collected health Data (RECORD) Statement [16].

### Data source

This study is based on the anonymized national hospital discharge records (HDRs), validated and processed by General Direction of health planning—Office 6 of the Ministry of health [17] and made available yearly by the Italian Ministry of Health to the Italian National Institute of Health (Istituto Superiore di Sanità—ISS) for epidemiological purposes. HDRs contain administrative, demographic, and clinical information on almost all hospitalizations in Italy, covering up to 99% of admissions at the national level [17]. A maximum of one main + 10 secondary procedures and one main + 5 secondary diagnoses are recorded by the International Classification of Diseases, 9th revision—Clinical Modification (ICD9-CM).

### Data extraction and processing

The dataset includes all HDRs between 1 January 2001, and 31 December 2023. The following procedure ICD9-CM code was considered of interest for the study: 81.51 “Total hip replacement.” All records reporting such code at least once, either as the principal or a secondary procedure, and not reporting urgency diagnosis codes (820.xx, “Fracture of neck of femur”), either as the principal or a secondary diagnosis, were included in the analysis and classified as total hip replacement (THR).

Population data, stratified by age, were obtained from the Italian National Institute of Statistics (ISTAT) for the 2001–2024 period, with forecasting extending to 2050. Only the resident population aged 50 years or older was considered in the analysis, based on the observation of a minimal proportion of younger patients in THR data.

### Regression-based forecasting of future THR volumes

The study applied six alternative statistical models to assess trends and forecast future THR volumes in Italy. The reason behind the application of such a wide range of models relies in the will to make the results of the present work comparable with the major literature on the topic of forecasted surgery volumes for THAs. Indeed, the first four of the proposed models respect what was established as gold standard in the field to address the examined research question and report results methodologically comparable at the international level [7–12, 14, 18–25]. However, the last two reported models are new proposals for the forecasting of THA volumes, justified by the need for a relaxed hypothesis set. Indeed, such models do not require strict and unrealistic distributional and mathematical assumptions for their coherent application.

The models include logistic regression (LM), Poisson regression (PM), logarithmic regression (LogM), inverse/power regression (IP), Poisson log-normal regression (PLN) and hierarchical Poisson regression with temporal effects (HPTE).

Parameter estimation was performed using maximum likelihood (MLE) for the LM, PM, logM, IP, and PLN models, while Bayesian inference via Markov chain Monte Carlo (MCMC) was applied for HPTE, with uninformative prior distributions. 95% prediction intervals (PI_95%_) were estimated analytically for LogM, by simulation for LM, PM, IP, and PLN and by MCMC estimation of the posterior predictive distribution of data for HPTE. Specifically, HPTE was implemented using the rstan package, the R interface to the Stan probabilistic programming language, which performs Hamiltonian Monte Carlo (HMC), a gradient-based MCMC algorithm that employs the No-U-Turn Sampler (NUTS) for efficient exploration of the posterior. Model-based forecasts for THA volumes until 2050 were computed for each model, and prediction intervals were estimated to quantify uncertainty. Increase and decrease rates are reported in terms of rate ratios (RR) with 95% prediction intervals (PI_95%_).

For the sake of reproducibility and mathematical rigor, a detailed description of statistical techniques and mathematical modeling formulation was reported in the Appendix: methodological considerations and details on model assessment.

### Model performance assessment

To ensure a wide evaluation of model performances, while capturing both the accuracy and the stability of model predictions, the following error metrics were computed: mean squared error (MSE), root mean squared error (RMSE), and mean absolute error (MAE). In addition, the standard deviation of squared errors (SD-SE) and the interquartile range of squared errors (IQR-SE) were included to assess the variability of errors and the potential influence of outliers. Last, the RMSE and MAE were normalized by the scale of the data providing a relative measure of error magnitude.

Data post-2019 were excluded from model training to minimize the impact of coronavirus disease-19 (COVID-19)-related distortions. Indeed, in 2020, elective surgeries were suspended and postponed in Italy to avoid the spreading of COVID-19 during the pandemic. However, data after 2020 were used for a second out-sample validation phase in a real-world setting. Goodness of fit was first assessed by using error metrics computed on the entire training dataset. Then, predictive performance was evaluated through an internal validation step using a rolling-origin approach. Finally, an additional evaluation phase was conducted to further assess predictive performance using out-of-sample data. In this phase, each model was trained on the entire dataset between 2001 and 2019. The models’ results were then validated by comparing their predictions against observed data from the year 2020 to 2023. These two steps provided real-world assessment of the models’ predictive abilities and allowed for an independent verification of their performance on data not involved in the model fitting or internal validation, also allowing for checking possible overfitting.

Data management and statistical analysis were performed by using the software R, version 4.3.0 (2023–04-21 ucrt)—“Already Tomorrow,” and Stan, version 2.32.2 accessed via the rstan R package.

## Results

**Fig. 1:**
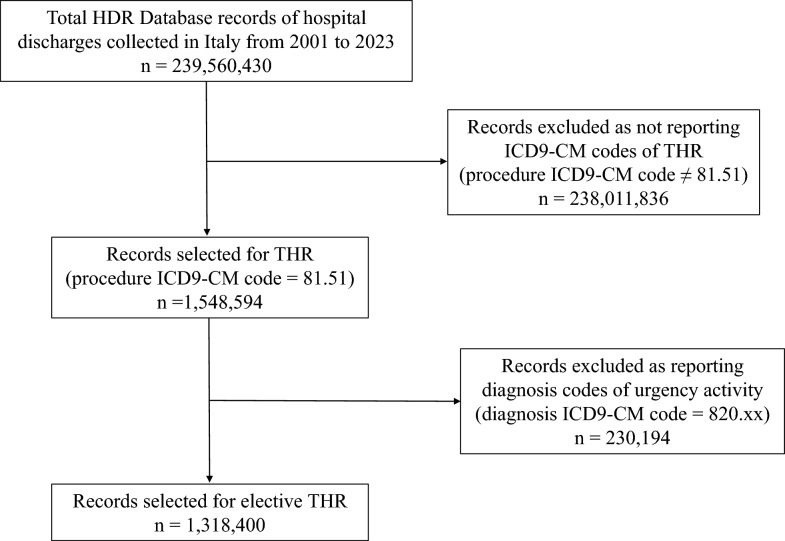
Flow chart of the data extraction process from the National hospital discharge records (HDR) database (2001–2023)

Figure 1 shows the results of the data extraction process from the 239,560,430 records included in the National HDR database from 2001 to 2023. By applying the selection criteria, 1,318,400 records for primary THAs performed in Italy between 2001 and 2023 were extracted. In total, 731,200 (55.6%) females and 587,190 (45.4%) males underwent THA in the observed 23 years, mostly between 65 and 74 years (472,246 cases, 35.7%). Demographics of patients that had undergone THA in the observed period are reported in Table 1.

**Table 1:** Demographics of patients

	Females		Males		Total	
Age classes	N	*%*	N	*%*	N	%
< 45	19,954	*2.7*	31,038	*5.3*	50,992	3.9
45–54	52,459	*7.2*	71,144	*12.1*	123,603	9.4
55–64	132,230	*18.1*	142,807	*24.3*	275,037	20.9
65–74	267,700	*36.6*	204,546	*34.8*	472,246	35.7
75–84	230,768	*31.6*	126,125	*21.5*	356,893	27.1
> 84	28,099	*3.8*	11,530	*2*	39,629	3
Total	731,200	*100*	587,190	*100*	1,318,400	100

**Fig. 2:**
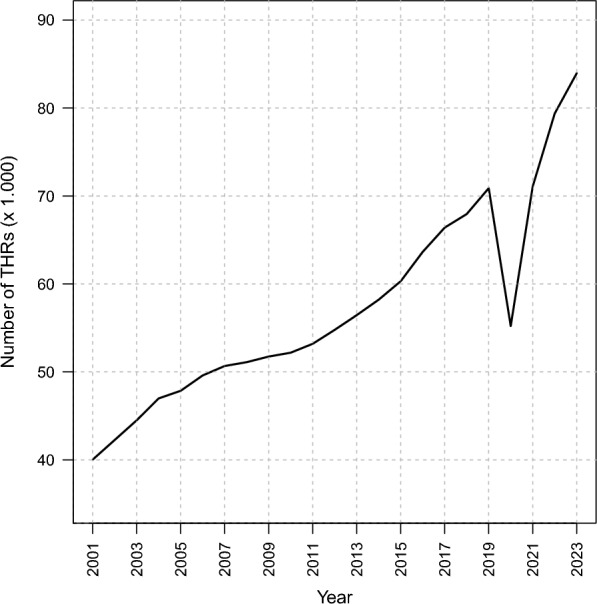
Counts of elective total hip replacements (THRs) performed by year. Data Sources: Ministry of Health, National HDR database (2001–2023)

The total number of surgeries increased by approximately 80%, rising from 68.270 in 2001 to 122.777 in 2023. In 2020, there was a decrease in the number of procedures performed (−16% compared with 2019), followed by a rapid recovery in the subsequent years (Fig. 2).

### Forecasting

**Fig. 3:**
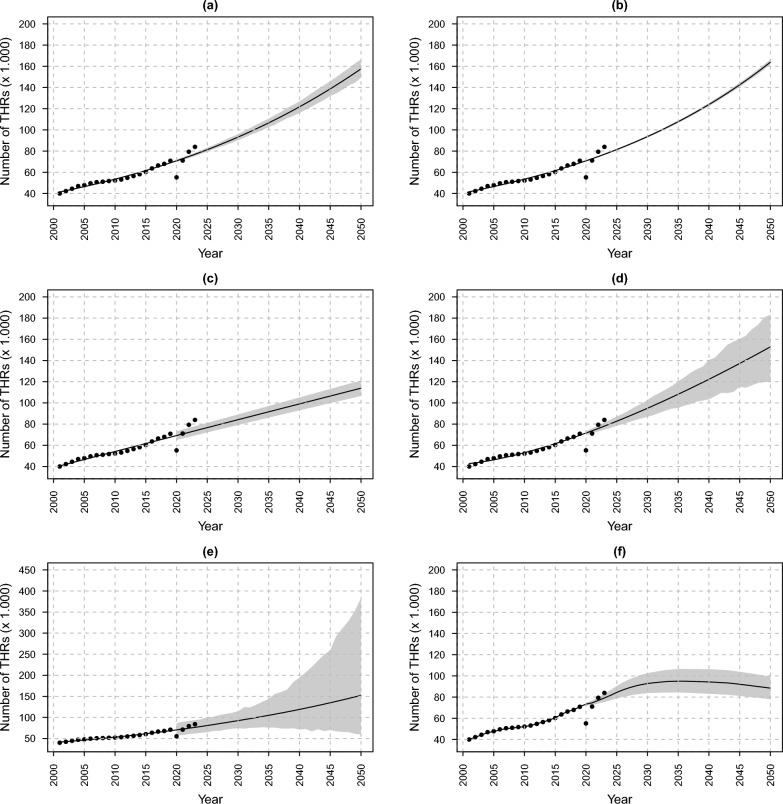
Historical (2001–2019) and forecasted (2020–2050) volumes and 95% prediction intervals of HRs: **a** logistic regression (LM), **b** Poisson regression (PM), **c** logarithmic regression (LogM), **d** inverse/power regression (IP), **e** Poisson log-normal regression (PLN), and **f** hierarchical Poisson regression with temporal effects (HPTE). Data sources: Ministry of Health, National HDR database (2001–2023); National Institute of Statistics, population data (2001–2024) and forecasts (2025–2050)

LM (Fig. 3a), PM (Fig. 3b), IP (Fig. 3d), and PLN (Fig. 3e) exhibit moderate growth trends, with expected RR of 2.2 (PI_95%_: 2.1; 2.3), 2.3 (PI_95%_: 2.3; 2.3), 2.2 (PI_95%_: 1.7; 2.6), and 2.1 (PI_95%_: 0.8; 5.4), respectively, by 2050 compared with 2019. LogM (Fig. 3c) predicts a lower growth, with expected increase with RR equal to 1.6 (PI_95%_: 1.5; 1.7). HPTE (Fig. 3f) provides the most conservative forecasts, resulting in an overall forecasted increase of 1.2 (PI_95%_: 1.1; 1.4) in terms of RR between 2019 and 2050. Moreover, the HPTE model predicts moderate growth until 2036, equal to 1.3 (PI_95%_: 1.1; 1.4), followed by a decline until 2050, diverging from the other models that anticipate a continuous growth. Supplementary Materials Table A1 reports details on actual, fitted, and forecasted volumes with prediction intervals on which Fig. 3 is built.

### Model assessment

The overall error metrics exhibit variation across models, with HPTE consistently outperforming all the others in terms of both accuracy (lower MSE, RMSE, and MAE) and error stability (lower SD-SE and IQR-SE). In terms of relative error ratios (ratio RMSE and ratio MAE), predictions carried out via HPTE are substantially the most precise, related to the scale of the problem, while the other models tend to produce errors of a comparable magnitude to the observed data. Indeed, RMSE and MAE ratio range from 2.3 to 3.1 for all models but HPTE, which achieves substantially lower relative errors equal to 0.42 and 0.32, respectively (Table 2).

**Table 2:** Models’ performance metrics on the training set (2001–2019)

	MSE	RMSE	MAE	SD-SE	IQR-SE	Ratio RMSE	Ratio MAE
LM	1,937,682	1,392	1,269	1,332,900	2,620,715	2.57	2.34
PM	1,901,699	1,379	1,264	1,289,012	2,397,894	2.55	2.33
LogM	2,799,160	1,673	1,460	2,621,583	3,055,782	3.09	2.70
IP	2,035,286	1,427	1,267	1,849,011	1,528,716	2.64	2.34
PLN	1,952,445	1,397	1,274	1,467,331	1,786,940	2.58	2.35
HPTE	50,486	225	173	71,008	53,728	0.42	0.32

**Fig. 4:**
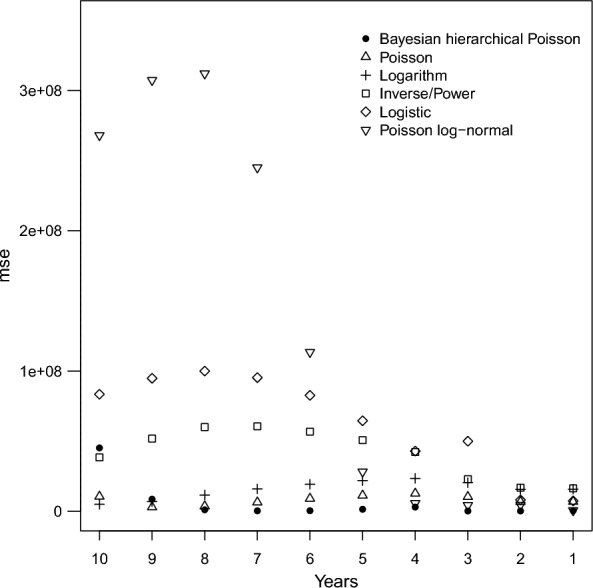
Mean squared error (MSE) for different models over the rolling-origin validation process. Data sources: Ministry of Health, National HDR database (2001–2019); National Institute of Statistics, population data (2001–2019)

The internal validation step by rolling-origin approach showed a progressive decrease in MSE as the training window expands across all models (Fig. 4). Definitely, HPTE is confirmed as the best performing model overall, according to the selected metrics. Further considerations on the internal validation process are reported in Appendix A.

### Out-of-sample validation

The error metrics for the out-of-sample validation phase (2020–2023) exhibit a slightly different pattern compared with the in-sample validation phase, with variations across models. In the first validation period (2020–2021), the LogM model is the most accurate model, while HPTE performs the worst. In contrast, HPTE emerges as the best model for the 2022–2023 period, consistent with its performance in the in-sample phase, showing both high predictive accuracy and error stability. Notably, when comparing all model performances across the two validation periods, errors are larger in 2020–2021 than in 2022–2023 (Table 3).

**Table 3:** Models’ performance metrics on two validation sets: years 2020–2021; years 2022–2023

	2020–2021			2022–2023		
	MSE	SD-SE	IQR-SE	MSE	SD-SE	IQR-SE
LM	123,604,525	170,508,154	120,567,472	34,528,513	20,167,881	14,260,846
PM	122,745,145	169,461,044	119,827,053	34,473,826	19,933,763	14,095,299
LogM	97,770,784	138,045,473	97,612,890	78,530,524	38,022,189	26,885,747
IP	136,525,801	183,604,543	129,828,018	23,295,317	15,517,839	10,972,769
PLN	116,792,805	162,961,421	115,231,126	40,633,850	22,077,840	15,611,390
HPTE	171,794,624	225,737,796	159,620,726	14,513,720	11,909,912	8,421,579

## Discussion

This study focused on examining the temporal trends of elective total hip arthroplasty procedures in Italy from 2001 to 2023, with the aim to forecast THR volumes up to 2050. The overall trend in THR procedures highlights a steady increase over time of +80%, with a significant drop in 2020 likely attributable to the impact of the COVID-19 pandemic. However, the rapid recovery observed in the following years suggests a strong rebound effect, indicating that postponed surgeries were largely rescheduled rather than permanently reduced. The volume of elective THRs is expected to increase between 10% and 40% by 2050.

### Forecasting evaluation and model choice

To achieve accurate forecasting, the behaviors of a wide range of models were compared. Most models predicted a continued increase, but with different growth rates and levels of uncertainty. LM, PM, IP, and PLN showed forecasted increases equals to 2.2 (PI_95%_: 2.1; 2.3), 2.3 (PI_95%_: 2.3; 2.3), 2.2 (PI_95%_: 1.7; 2.6), and 2.1 (PI_95%_: 0.8; 5.4), respectively, between 2019 and 2050. However, PLN exhibits wide prediction intervals, indicating significant uncertainty. This behavior aligns with findings from the predictive performance in evaluation phase, confirming its instability in long-term forecasting scenarios. LogM, in contrast, projects a lower increase of 1.6 (PI_95%_: 1.5; 1.7). HPTE provides the most conservative forecasts, with a moderate rise until 2036 and stabilization until 2050, which might reflect shifts in demographic changes affecting THR demand. The HPTE model outperforms all the other models in terms of error, fitting, stability, and dispersion, according to the considered metrics, reinforcing its reliability. These findings suggest that the HPTE model best fits the training data, minimizing both absolute and relative errors. Its superior performance may be attributed to its ability to better capture the underlying patterns in the training set. The only exception is the 2020–2021 validation period, where HPTE does not emerge as the best model. However, the results for this period should be interpreted with caution owing to the potential bias introduced by the impact of the COVID-19 pandemic. Indeed, errors are larger in 2020–2021 compared with 2022–2023, possibly reflecting the greater challenge of capturing trends during the initial phase of the pandemic, followed by a stabilization in subsequent years. These observations confirm the robustness of the HPTE model, as its error magnitude remains the lowest even when tested on entirely new data, showing superior predictive accuracy and stability, reinforcing its reliability in real-world predictive settings as providing realistic long-term projections. Then, its ability to capture historical trends while avoiding unrealistic growth assumptions might make it a valuable tool for healthcare planning and policy-making in the analyzed scenario.

### Clinical and health considerations

Future forecasts provide insights into the expected evolution of THR volumes over the coming decades (between +10% and +40% of yearly THRs by 2050). Indeed, projections of future volumes of primary joint arthroplasty, including THA, are valuable tools for orthopedic health policy planning. Therefore, the choice of providing evidence by a wide range of models highlights the will to make the provided results methodologically comparable to the most relevant international literature. Indeed, while some forecasting studies significantly overestimated growth rates [18, 19], other analyses using refined estimation models [14, 20] consistently report a year-on-year increase in arthroplasty procedures. This trend is primarily driven by population aging, rising obesity prevalence, and expanded surgical indications. Similar patterns have been observed in the USA, Australia, and Europe [14, 21–24]. These projections have proven to be reliable approximations of actual joint replacement utilization, supporting the application of modeling techniques based on historical trends and demographic projections to estimate future surgical demand and guide health system planning [25].

However, these projections must be interpreted along with the current challenges that orthopedic healthcare delivery in Europe is facing. The demand for orthopedic specialists in Europe is escalating due to population aging, rising rates of musculoskeletal disorders, and the backlog of elective surgeries postponed during the COVID-19 pandemic. However, this surge in demand is juxtaposed with a significant shortage of orthopedic surgeons, being a field where professionals require extensive training and experience. Those factors constitute a trend which is expected to worsen in the coming years [26]. Italy is currently facing a significant shortage of orthopedic surgeons, exacerbated by the aging of the medical workforce and insufficient generational replacement. Data shows that Italy has the oldest medical workforce in the EU, with 55.2% of physicians aged over 55 years and approximately 25% over 65 years, the highest percentage in Europe. The European Commission’s Joint Research Centre projections indicate that the EU as a whole will require a 30% increase in the number of doctors by 2071 to meet healthcare demands, with particular urgency in specialties such as orthopedics, while Italy’s situation presents even more critical challenges given its demographic profile [27].

The implications are significant for resource allocation, workforce planning, and infrastructure development. Without proactive and targeted strategies, healthcare systems may face mounting pressure in terms of surgical throughput, inpatient capacity, rehabilitation services, and overall financial sustainability. Substantial investment in arthroplasty services will be necessary to maintain service levels amid the growing procedural volume.

The main limitation of this study is the potential bias introduced by the administrative nature of ICD9-CM coding system. Coding errors, wrongly reported codes, and missing codes were possible. However, Italian HDRs were proven to correctly identify primary hip arthroplasty and elective causes of intervention, as required for the present study, in over 95% of the cases [28], confirming the reliability of used data. Nonetheless, information on pre- and postoperative and on implanted devices, useful for complete assessments and thorough clinical- and health-related decision making, is not covered in HDRs. This issue underlines the need to promote participation in the Italian Arthroplasty Registry (RIAP). Although RIAP has been collecting data for hip prostheses since 2007, completeness in data was on average close to 40% over the collection years [29]. Indeed, nowadays, participation in RIAP is still voluntary and is based on specific agreements signed between Italian Regional Health Systems and the ISS. This study and the landscape of the Italian context depicted want to encourage surgeons’ participation in RIAP, as the Italian registry still does not align to other structured registries at the international level in terms of completeness and coverage [30]. Second, 2020 and 2021 data are influenced by the exceptional impact of the COVID-19 pandemic, which significantly disrupted healthcare systems and patient mobility. As a result, the errors computed for these years may represent an overestimation of the predictive discrepancies that would have been observed in the absence of the pandemic. This should be taken into account when interpreting the out-of-sample validation results. Third, additional variability might be introduced by the population data forecasts by ISTAT, with subsequent increased predictive uncertainty and wider prediction intervals for the provided forecasts. Last, the age threshold used to identify the target population used in the modeling process is arbitrary and different choices may lead to different outcomes. In the present study, the choice of such threshold takes into account population dynamics and aging occurred in Italy for the last 20 years.

## Conclusions

This study reinforces previous evidences indicating that the global demand for THA, including in Italy, is expected to continue rising over the coming decades, primarily driven by an aging population. This forecasted increase is likely to impose a substantial strain on future healthcare systems. However, shifts in lifestyle habits and advancements in therapeutic options for joint diseases may help mitigate the trend, potentially easing the pressure on healthcare services.

It is important to note that the accuracy of such projections may be affected by various unforeseeable variables; therefore, ongoing short-term monitoring could enhance the reliability and practical value of these forecasts.

## Supplementary Information

Supplementary Material 1.

## Data Availability

The data that support the findings of this study were provided from The Italian Ministry of Health to the Italian National Institute of Health but restrictions apply to the availability of these data, which were used under license for the current study, and so are not publicly available. Data are however available on aggregated form from the authors upon reasonable request and with permission of the Italian National Institute of Health.

## Methodological considerations and details on model assessment

For the sake of reproducibility and mathematical rigor, the present appendix reports a detailed description of statistical techniques and mathematical modeling formulation. Moreover, details on the error metrics used to evaluate model performance both on training and validation data are provided.

Each model is defined by the following, where *y* represents the THR volume, *t* denotes the year and Pop refers to the resident population aged 50 years or older:

1. Logistic regression (LM): $y\left(t\right)=\frac{A}{\left(1+{e}^{\frac{\alpha -t}{\beta }}\right)}+{\varepsilon }_{t}$, with ${\varepsilon }_{t}\sim N\left(0,{\sigma }^{2}\right)$
2. Poisson regression (PM): $y\left(t\right)\sim Poisson\left({e}^{\lambda \left(t\right)}\right),$ where $\lambda \left(t\right)=\alpha +\beta t$
3. Logarithmic regression (LogM): $y\left(t\right)=\alpha +\beta ln(t)+{\varepsilon }_{t}$, with ${\varepsilon }_{t}\sim N\left(0,{\sigma }^{2}\right)$
4. Inverse/power regression (IP): $y\left(t\right)=\alpha +\frac{\beta }{{t}^{\gamma }}+{\varepsilon }_{t}$ with ${\varepsilon }_{t}\sim N\left(0,{\sigma }^{2}\right)$
5. Poisson log-normal regression (PLN): $y\left(t\right)|\lambda (t)\sim Poisson\left({e}^{\lambda \left(t\right)}\right)$ With $\Lambda =\left(\lambda \left(1\right),\dots ,\lambda \left(T\right)\right)\sim N\left(X{\rm B},\sum \right)$;
  $X$ is a $T\times 3$ design matrix containing the predictors: $X\left(t\right)=\left(1,Pop(t),t\right)$;
  ${\rm B}$ is a $3\times 1$ vector of regression coefficients: ${\rm B}=\left(\alpha ,{\beta }_{1},{\beta }_{2}\right)$.
  ∑ is a full covariance matrix describing the correlation structure among the latent variables $\lambda (t)$
6. Hierarchical Poisson regression with temporal effects (HPTE): $y\left(t\right)|\lambda (t)\sim Poisson\left({e}^{\lambda \left(t\right)}\right)$, where $\lambda \left(t\right)=\alpha +{\beta }_{1}Pop\left(t\right)+\theta (t)$.
  $\theta (t)$ represents the temporal random effect, modeled using an autoregressive process of order 1 (AR(1)):
  $$\theta \left(t\right)=\varphi \theta \left(t-1\right)+{\sigma }_{\theta }\varepsilon \left(t\right), \varepsilon \left(t\right)\sim N\left(\text{0,1}\right) \forall t>1$$
  And $$\theta \left(1\right)={\sigma }_{\theta }\varepsilon \left(1\right), \varepsilon (1)\sim N\left(\text{0,1}\right)$$

95% Prediction intervals for the future forecasts of the 6 models were computed as follows. LM: samples of the vector of parameters were drawn from a multivariate normal distribution, centered on the estimated vector and with variance–covariance structure approximated using the inverse of the hessian matrix; for each sample of the vector of parameters one sample of the counts was drawn according to the model formulation and adding a Gaussian random error to account for the variability of the data; empirical quantiles of this distribution of counts were computed. PM: samples of the vector of parameters were drawn from a multivariate normal distribution centered on the estimated vector with estimated variance–covariance structure; for each sampled vector of parameters one sample of the counts was drawn from the Poisson distribution; empirical quantiles of this distribution of counts were computed. LogM: analytical definition of the intervals as y ^ ± t_(n-p,1-α/2) √(VAR[Y ^]) were used, where y ^ denote the prediction and VAR[Y ^] indicate the total variance of the predictive distribution, being equal to the sum of the estimated variance of the parameters and of the estimated variance of the data. IP: samples of the vector of parameters were drawn from a multivariate normal distribution, centered on the estimated vector and with variance–covariance structure approximated using the inverse of the hessian matrix; for each sample of the vector of parameters one sample of the counts was drawn according to the model formulation and adding a Gaussian random error to account for the variability of the data; empirical quantiles of this distribution of counts were computed. PLN: PLN models were fitted on bootstrapped samples of the data and the corresponding parameter estimates were extracted; the variance–covariance matrix for the vector parameters were estimated based on the empirical distribution of the samples; the parameters were drawn from a multivariate normal distribution centered on the original estimates and with the estimated variance–covariance structure; for each sampled vector of parameters one sample of the counts was drawn from the Poisson distribution; empirical quantiles of this distribution of counts were computed. HPTE: the posterior predictive distribution of the counts was approximated extracting the posterior samples of the model parameters and computing, for each of these samples, the corresponding prediction according to the model definition; the empirical quantiles of this distribution were computed.

For sake of thoroughness, we report the formulas of the error metrics used to evaluate model performance both on training and validation data. These include measures of accuracy—Mean Squared Error (MSE), Root Mean Squared Error (RMSE), and Mean Absolute Error (MAE)—as well as metrics of error variability—Standard Deviation of Squared Errors (SD-SE) and Interquartile Range of Squared Errors (IQR-SE):

$$MSE=\frac{1}{T}\sum_{t=1}^{T}{\left(y\left(t\right)-\hat{y}(t)\right)}^{2}$$

$$RMSE=\sqrt{\frac{1}{T}\sum_{t=1}^{T}{\left(y\left(t\right)-\hat{y}(t)\right)}^{2}}$$

$$MAE=\frac{1}{T}\sum_{t=1}^{T}\left|y\left(t\right)-\hat{y}(t)\right|$$

$$SD-SE=\sqrt{\frac{1}{T-1}\sum_{t=1}^{T}\left[{\left(y\left(t\right)-\hat{y}(t)\right)}^{2}-MSE\right]}$$

$$IQR-SE={Q}_{3}\left({\left(y\left(t\right)-\hat{y}(t)\right)}^{2}\right)-{Q}_{1}({\left(y\left(t\right)-\hat{y}(t)\right)}^{2})$$

$$RatioRMSE=\frac{RMSE}{mean(y\left(t\right))}$$

$$RatioMAE=\frac{MAE}{mean(y\left(t\right))}$$

where $y(t)$ are the observed values, $\hat{y}(t)$ are the corresponding model predictions, ${Q}_{1}$ and ${Q}_{3}$ denote, respectively, the first and third quartiles of the distribution of the squared errors ${\left(y\left(t\right)-\hat{y}(t)\right)}^{2}$, corresponding to the 25th and 75th percentiles.

The proposed rolling-origin validation scheme is conducted mimicking real-world forecasting scenarios. Specifically, the dataset was iteratively split into a training and a validation set, where the training set included data up to a given year $t$, and the validation set consisted of data from $t$ +1 onward. The model was fitted on the training set, and the mean squared error (MSE) was computed on the validation set. The process started with a training set containing data from 2001 to 2009 and a validation set from 2010 to 2019. The procedure was then repeated iteratively, extending the training set by one year each time and adjusting the validation set accordingly (e.g., training up to 2010, validating on 2011–2019; training up to 2011, validating on 2012–2019, and so on) until the final split, where the training set included data up to 2018 and validation was performed solely on 2019. The validation procedure allows for a robust assessment of the model’s predictive performance over time and validates model performances when the length of observed and forecasted time windows expand and narrow.

When leaving out 10–9 years, PLN exhibits the highest MSE. In contrast, LogM and PM deliver the most accurate predictions. HPTE suddenly catches up with the best models. When leaving out 8–6 years ahead, PLN keep showing the highest errors, while HPTE surpasses LogM and PM. In the final phase, leaving 5–1 years out, PLN performances become comparable to the others and ultimately ranks as the second best performer, beaten only by HPTE, which consistently emerges as the best model (Fig. 4). The models with higher MSE values, exhibit greater variability across iterations, indicating instability in their predictions. When the training window is small, PLN struggles significantly, likely due to its higher number of parameters. In contrast, the LogM and PM perform the best, confirming that simpler models with fewer parameters tend to be more robust in low-data scenarios. The performance of PLN gradually improves as the training window expands, emerging as one of the top performers in the final validation phases. Nonetheless, PLN exhibits the widest prediction intervals, indicating greater uncertainty in future estimates (Fig. 3e). For the tested models, the progressive definitive decrease in MSE as the training window expands is shown in Fig. 4. This aligns with expectations: incorporating more historical data improves parameter estimation and predictive accuracy. Notably, the HPTE model, despite having a large number of parameters, almost immediately reaches and even surpasses the performance of the simpler models. Overall, the analysis highlights the strengths and limitations of different predictive models for ETHRs. While simpler models provide stable forecasts, they may fail to capture long-term complexities, whereas other models can exhibit excessive variability.

## Abbreviations

THA/THR: Total hip arthroplasty/Total hip replacement
HDR: Hospital discharge record
ISS: Istituto Superiore di Sanità—Italian National Institute of Health
ICD9-CM: International Classification of Diseases, 9th Revision, Clinical Modification
LM: Logistic regression
PM: Poisson regression
LogM: Logarithmic regression
IP: Inverse/power regression
PLN: Poisson log-normal regression
HPTE: Hierarchical Poisson regression with temporal effects
MLE: Maximum likelihood estimator
MCMC: Markov chain MonteCarlo
HMC: Hemiltonian MonteCarlo
NUTS: No-u-turn sampler
RR: Rate ratio
PI: Prediction interval
MSE: Mean square error
RMSE: Root MSE
MAE: Mean absolute error
SD-SE: Standard deviation of squared errors
IQR-SE: Interquartile range of squared errors
RIAP: Registro Italiano ArtroProtesi—Italian Arthroplasty Registry
